# PREDICTIVE MODELING OF HEALTH STATUS OVER LIFESPAN BASED ON SENESCENCE BIOMARKERS

**DOI:** 10.1093/geroni/igae098.3691

**Published:** 2024-12-31

**Authors:** Lingzi Tang, Siarhei Hladyshau, Allison Ross, Kirsten Nyrop, Amy Entwistle, Hyman Muss, Denis Tsygankov

**Affiliations:** Georgia Institute of Technology, Atlanta, Georgia, United States; St. Jude Children’s Research Hospital, St. Jude Children’s Research Hospital, Tennessee, United States; University of North Carolina, Chapel Hill, North Carolina, United States; UNC Medicine, Chapel Hill, North Carolina, United States; Sapere Bio, Durham, North Carolina, United States; University of North Carolina - Chapel Hill, Pittsboro, North Carolina, United States; Georgia Institute of Technology and Emory University, Atlanta, Georgia, United States

## Abstract

Cellular senescence is known to play a significant role in aging and the development of age-related diseases. However, the complex interplay between the accumulation and clearance of senescent cells and the relationship of these processes to the physiological decline during natural aging is not understood. Here, we report a study that explores the potential of cellular and immune senescence biomarkers (p16, p14, LAG3, CD28, and CD244) in predicting health metrics as determined by standard clinical assessments. Data were collected from a cohort of 250 healthy participants aged 25 to 85 years and included gene expression in T-cells from peripheral blood, comprehensive blood panels, quality of life (QOL) surveys, body composition analysis, and physical performance evaluations. A combination of computational techniques, including correlation analysis, unsupervised hierarchical clustering, and machine learning techniques (such as an ensemble of bagged decision trees), was employed to assess the predictive capabilities of the biomarkers relative to the health assessments and vice versa. Our findings indicate that blood and QOL assessments generate distinct participant clusters, both strongly associated with p16 expression. The highest accuracy in predicting p16 expression was achieved by integrating measurements across various assessments, suggesting a systemic role for this biomarker. Furthermore, our analysis led to a scoring system that produces integrative metrics strongly and specifically correlating with different senescence biomarkers, suggesting a simple, practical method to differentiate between varying levels of cellular and immune senescence in naturally aging individuals.

